# Neutrophil-like Monocytes Increase in Patients with Colon Cancer and Induce Dysfunctional TIGIT+ NK Cells

**DOI:** 10.3390/ijms25158470

**Published:** 2024-08-02

**Authors:** Alessia Calabrò, Fabiana Drommi, Giacomo Sidoti Migliore, Gaetana Pezzino, Grazia Vento, José Freni, Gregorio Costa, Riccardo Cavaliere, Irene Bonaccorsi, Mariagrazia Sionne, Stefania Nigro, Giuseppe Navarra, Guido Ferlazzo, Claudia De Pasquale, Stefania Campana

**Affiliations:** 1 Laboratory of Immunology and Biotherapy, Department Human Pathology “G. Barresi”, University of Messina, Via Consolare Valeria 1, 98125 Messina, Italy; calabroalessia@hotmail.it (A.C.); drommifabiana@gmail.com (F.D.); tpezzino@unime.it (G.P.); gregorio.costa@unime.it (G.C.); ibonaccorsi@unime.it (I.B.); cdepasquale@unime.it (C.D.P.); scampana@unime.it (S.C.); 2 Translational Immunobiology Unit, Laboratory of Infectious Diseases, National Institute of Allergy and Infectious Diseases, National Institutes of Health, BLDG 50, RM 6308, Bethesda, MD 20892, USA; giacomo.sidotimigliore@nih.gov; 3 Department of Experimental Medicine (DIMES), University of Genoa, Via Leon Battista Alberti 2, 16132 Genova, Italy; grazia.vento@edu.unige.it; 4 Laboratory of Histology, Department of Biomedical, Dental, Morphological and Functional Imaging Sciences, University of Messina, Via Consolare Valeria 1, 98125 Messina, Italy; jofreni@unime.it; 5 Clinical Pathology Unit, University Hospital Policlinico G. Martino, 98125 Messina, Italy; rcavaliere@unime.it; 6 Oncologic Surgery, Department of Human Pathology of Adult and Evolutive Age, University of Messina, Via Consolare Valeria 1, 98125 Messina, Italy; mariagrazia.sionne92@libero.it (M.S.); nigrostefania23@gmail.com (S.N.); giuseppe.navarra@unime.it (G.N.); 7 Unit of Experimental Pathology and Immunology, IRCCS Ospedale Policlinico San Martino, Largo R. Benzi 10, 16132 Genova, Italy

**Keywords:** MDSCs, monocytes, neutrophil-like cells, NK cells, TIGIT, CRC, human

## Abstract

Myeloid-derived suppressor cells (MDSCs) are a heterogeneous family of immune cells including granulocytic (CD14neg/CD15+/HLA-DRneg) and monocytic subtypes (CD14+/CD15neg/HLA-DRneg). In the present study, we found a population of monocytes expressing the granulocyte marker CD15 that significantly increased in both peripheral blood (PB) and tumoral tissues of patients with colorectal cancer (CRC). Further phenotypical analysis confirmed the granulocytic-like features of this monocyte subpopulation that is associated with an increase in granulocyte–monocyte precursors (GMPs) in the PB of these patients (pts). Mechanistically, this granulocyte-like monocyte population suppressed NK cell activity by inducing TIGIT and engaging NKp30. Accordingly, an increased frequency of TIGIT+ NK cells with impaired functions was found in both the PB and tumoral tissue of CRC pts. Collectively, we provided new mechanistic explanations for tumor immune escape occurring in CRC by showing the increase in this new kind of MDSC, in both PB and CRC tissue, which is able to significantly impair the effector functions of NK cells, thereby representing a potential therapeutic target for cancer immunotherapy.

## 1. Introduction

Cancer has evolved different mechanisms to evade the host’s immune response, including the accumulation of immunosuppressive cells [1,2,3,4]. Among these, MDSCs are cells of myeloid origin that, through several mechanisms, can inhibit the immune response and support tumor growth [5,6,7,8].

MDSCs represent a heterogeneous family of immune cells distinguished according to their origin: granulocytic MDSCs (G-MDSCs) defined as CD11b+CD33+CD14negCD15+CD66b+HLA-DRdim/neg; monocytic MDSCs (M-MDSCs) identified as CD11b+CD33+CD14+CD15negHLA-DRdim/neg [9,10].

The frequency of MDSCs widely varies in healthy donors and significantly increases in patients (pts) with cancers [11,12], accumulating in different compartments including tumor tissues, peripheral blood (PB) and bone marrow (BM) [13].

Recently, besides the two major groups of MDSCs, a new monocytic population expressing the granulocyte marker CD15 has been identified in pts with NSCLC [14] and melanoma [15]. Similarly, in the circulation of cancer pts, a monocyte subpopulation expressing CD66b, a marker typically expressed by neutrophils, has been identified [16]. This population comprises a subset showing both the CD15 marker and a low level of HLA-DR [16].

Although M-MDSCs and G-MDSCs differentiate as monocytes and neutrophils, respectively, increased production of tumor-released factors, such as GM-CSF, CSF-1 and other growth factors, can result in abnormal myelopoiesis responsible for the accumulation of a defined population of monocyte-like precursors of granulocytes (MLPGs) [17]. These MLPGs maintain their monocytic nature while acquiring the expression of different neutrophil genes and the capability to differentiate in neutrophils. Moreover, the same tumor-released growth factors, together with the selective increase in MLPGs, can induce, in various tumors, an increase in the upstream granulocyte–monocyte precursors (GMPs) skewed toward granulocytic differentiation [18].

Here, we observed an increase in a population of monocytes expressing CD15 within the M-MDSC compartment of pts with colon cancer (CRC) in both PB and tumor tissues. Phenotypical and morphological analysis revealed that these CD15+ monocytes share both monocytic and neutrophilic properties and are associated with an increase in circulating GMPs.

Moreover, we revealed a mechanism by which CD15+ monocytes could suppress NK cell antitumor functions by promoting the acquisition of the inhibitory receptor TIGIT. In light of the increased number of this new subset of immunosuppressive MDSCs in CRC, these cells can represent a promising therapeutic target for future immunotherapeutic strategies.

## 2. Results

### 2.1. Monocytes Expressing the Granulocytic Marker CD15 Increase in the Blood and Tumor Tissue of CRC Patients

**Figure 1:**
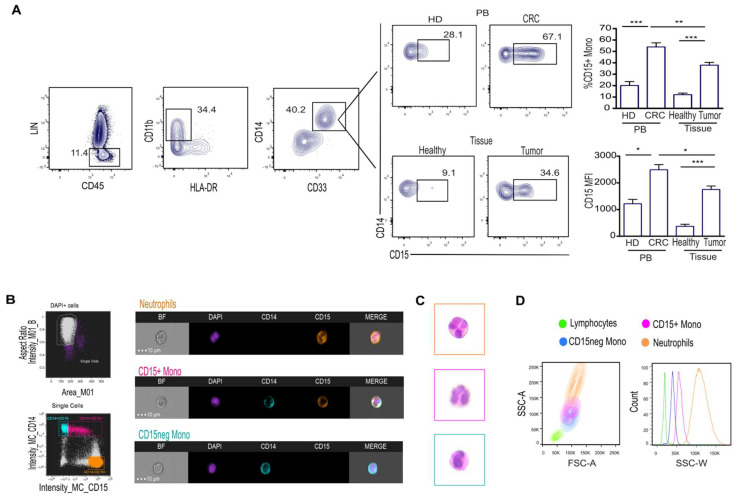
M−MDSCs expressing the granulocyte marker CD15 increase in the blood and tumor tissue of CRC pts. (**A**) Gating strategy used for identification of M-MDSCs (**left**) and representative dot plot showing CD15+ monocytes in PB of CRC pts, HD and tissues of CRC pts (**right**). Bars represent the frequency ±  SEM of CD15+ monocytes or the MFI of CD15 antigen (n = 30) * *p* < 0.05; ** *p* < 0.01; *** *p* < 0.001. (**B**) CD15 expression was assessed by imaging flow cytometry of FACS-sorted purified neutrophils and CD15+ and CD15neg monocytes from CRC pts. Comparative brightfield (BF), nuclear dye DAPI, CD14, CD15 and channel merge profile are shown. (**C**) Hematoxylin and eosin staining on CD15+, CD15neg monocytes and neutrophils from CRC pts. (**D**) Flow cytometer analysis of morphological parameters (**left**) and scatter width (**right**) of the indicated populations from CRC pts.

Over the course of our analysis of the M-MDCSs, defined as CD45+ LINneg (CD3, CD56, CD19) CD11b+CD33+CD14+HLA-DRdim/neg, in pts with CRC, we observed a significant increase in CD15+ monocytes in both the PB and tumor tissue (Figure 1A).

Considering the low overall cell number obtained from tissue specimens, circulating CD15+ monocytes from CRC pts were used for subsequent phenotypical and functional analyses.

To further investigate the co-expression of CD14 and CD15 on monocytes, morphological cell analysis was performed on CD15+ monocytes by using an ImageStream multispectral imaging cytometer, which allows for the simultaneous integration of both flow cytometric and morphological information. To this aim, we analyzed CD15+ monocytes, CD15neg monocytes and neutrophils from the whole blood of CRC pts.

Compared to CD15neg monocytes and granulocytes showing singular expression of CD14 and CD15, respectively, CD15+ monocytes co-expressed both markers (Figure 1B). Of note, the observation of nuclear morphology of CD15+ monocytes, by DAPI and hematoxylin and eosin staining, revealed a multi-lobular structure reminiscent of the nucleus in neutrophils rather than in monocytes (Figure 1B,C). Along the same line, analysis of side scatter pulse width, a parameter capable of estimating cell diameter, revealed that CD15+ monocytes display an intermediate size between neutrophils and monocytes (Figure 1D). Therefore, these data reveal a significant increase in CD14+ monocytes expressing the granulocytic marker CD15 in both the PB and tumor tissue of CRC pts.

### 2.2. CD15+ Monocytes Display Neutrophil-like Features and Are Associated with a Higher Frequency of Circulating Granulocyte–Monocyte Precursors

Considering the similarities observed between CD15+ monocytes and neutrophils, we extended the phenotypical analysis of these cells to other markers belonging to both monocytic and neutrophil lineages.

**Figure 2:**
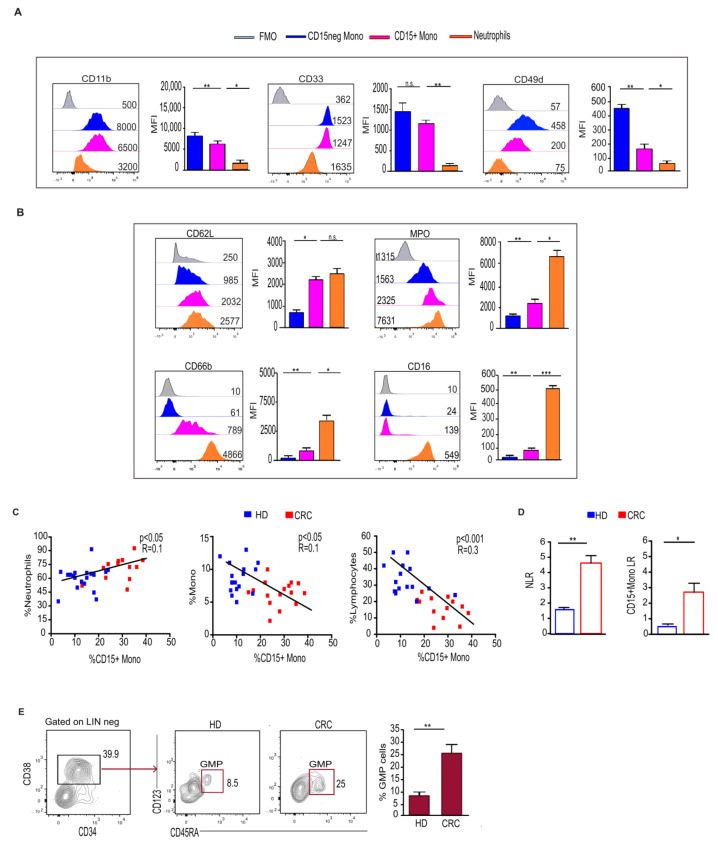
CD15+ monocytes display a granulocyte-like profile and are associated with an increase in circulating GMPs in CRC pts. (**A**,**B**) Histogram and relative statistical analysis showing the expression of the indicated markers associated with monocytic (**A**) and granulocytic (**B**) profiles, assessed on CD15+, CD15neg monocytes and neutrophils from CRC pts. Fluorescence minus one (FMO) staining was used as control. * *p* < 0.05; ** *p* < 0.01; *** *p* < 0.001. (**C**) Correlation between the frequency of CD15+ monocytes and neutrophils and monocytes and lymphocytes. (**D**) Bars represent the ratio between neutrophil-to-lymphocyte (NLR) or between CD15+ monocytes and lymphocytes (CD15+ monoLR) assessed on HD and CRC pts. * *p* < 0.05; ** *p* < 0.01. (**E**) Gating strategy used for identification of myeloid precursors and representative dot plot showing circulating granulocyte–monocyte progenitors (GMPs) in CRC pts (LIN neg: CD3, CD19, CD56). Bars represent the percentage ± SEM of GMPs (n = 5). ** *p* < 0.01. n.s.= not significant.

Our data revealed that CD15+ monocytes display high levels of CD33 and CD11b (Figure 2A), typical myeloid markers highly expressed on monocytes and present to a lesser extent on neutrophils [19]. CD49d, an integrin homogeneously expressed on monocytes but absent on mature neutrophils [20], is expressed at an intermediate level on CD15+ monocytes (Figure 2A). Of note, neutrophils can express this marker at an intermediate level during the immature stage [21,22]. CD15+ monocytes highly express typical neutrophil markers, such as CD62L and CD66b (Figure 2B), which are constitutively expressed by neutrophils but quite absent on monocytes [23,24]. The effector molecule MPO [25] is expressed by CD15+ monocytes at a higher level compared to monocytes, but it is similar to that observed in neutrophils (Figure 2B). Conversely, CD16, expressed in neutrophils [26], is completely absent on CD15+ monocytes, as well as on a large fraction of monocytes (Figure 2B).

Altogether, these findings suggest that CD15+ monocytes, sharing phenotypical features with both monocytes and neutrophils, can represent a monocytic subset with neutrophil-like features.

Consistent with this finding, the frequency of CD15+ monocytes is positively correlated with that of granulocytes but was inversely associated with that of monocytes and lymphocytes (Figure 2C). Moreover, a similar or higher neutrophil-to-lymphocyte ratio (NLR) was found in CRC pts compared to HD (Figure 2D). In contrast to this, we observed that the ratio of CD15+ monocytes-to-lymphocytes reached a higher value in pts with CRC (Figure 2D).

The increase in CD15+ monocytes with neutrophil features in CRC pts raised the question of whether circulating hematopoietic precursors exhibited myeloid bias with a skew toward granulocytic differentiation. To address this issue, we determined the frequency of granulocyte–monocyte progenitors (GMPs) within the LINneg/CD34+ population in the PB of CRC pts. We found a significant increase in circulating GMPs in CRC pts that might explain the increase in a monocyte population biased towards a granulocytic profile (Figure 2E).

### 2.3. CD15+ Monocytes Inhibit NK Cell Activity via TIGIT Induction and NKp30 Engagement

**Figure 3:**
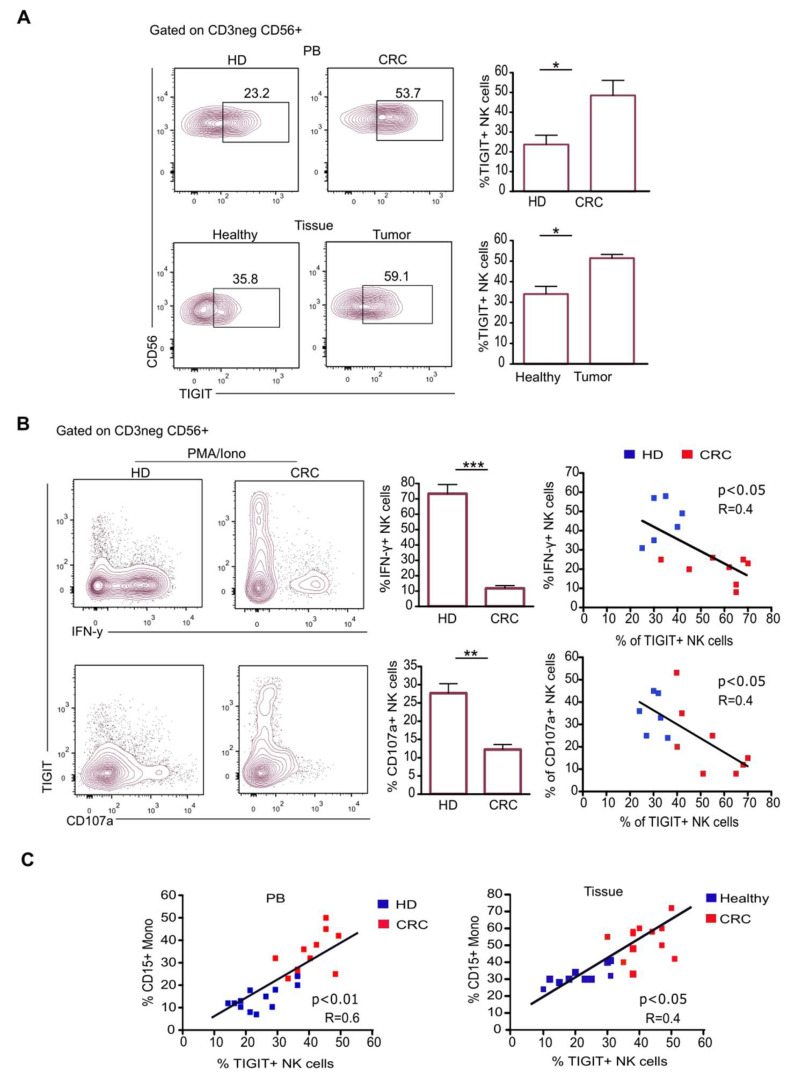
Frequency of dysfunctional TIGIT+ NK cells increases in CRC pts and correlates with that of CD15+ monocytes. (**A**) Representative dot plots and relative statistical analysis showing the expression of TIGIT on NK cells from PB of CRC pts and HD (upper panel) and tissues of CRC pts (lower panel). Bars represent MFI ± SEM of TIGIT+ NK cells (n = 12) * *p* < 0.05; (**B**) representative dot plots and relative statistical analysis showing the expression of CD107a and the production of IFN-γ by PMA/Iono-stimulated NK cells from CRC pts and HD with respect to TIGIT expression. Bars represent percentage ± SEM of CD107a+ and IFN-γ+ NK cells (n = 14) ** *p* < 0.01; *** *p* < 0.001; correlation between TIGIT+ NK cells and IFN-γ+ or CD107a+ NK cells from HD (blue) and CRC pts (red). (**C**) Correlation between the frequency of CD15+ monocytes and TIGIT+ NK cells in both PB and tissues.

In human CRC, tumor-infiltrating NK cells showed high TIGIT expression that is associated with their dysfunction [27]. Accordingly, we observed an increase in TIGIT expression on NK cells from both the PB and tumor compartments of CRC pts (Figure 3A). Remarkably, when analyzed for their effector functions, NK cells were impaired in both IFN-γ production and cytotoxicity (Figure 3B). We also observed that the percentage of dysfunctional TIGIT+ NK cells was positively correlated with that of CD15+ monocytes in both CRC-PB and tissues (Figure 3C), and therefore we asked whether this population could have a role in the induction of TIGIT in NK cells.

**Figure 4:**
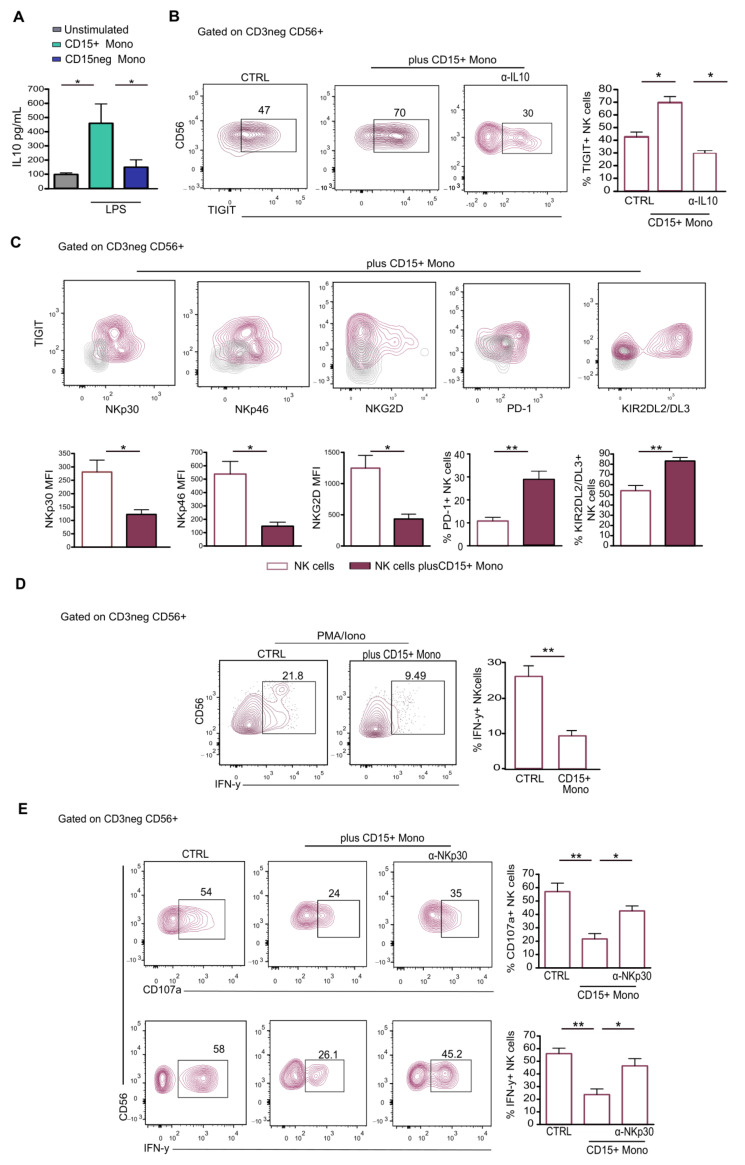
CD15+ monocytes induce dysfunctional NK cells via IL-10 and NKp30 engagement. (**A**) IL-10 concentration was measured in supernatants of CD15+ monocytes and CD15neg monocytes isolated from CRC pts (n = 4). Unstimulated monocytes were used as control. * *p* < 0.05. (**B**) Expression of TIGIT assessed on FACS-sorted NK cells from HD following 3 days of coculture with CD15+ monocytes from CRC pts in the presence of IL-10 blocking mAb. Bars indicate percentage ± SEM of TIGIT+ NK cells (n = 5) * *p* < 0.05. (**C**) Expression of activating receptors (NKp30, NKp46 and NKG2D) and inhibitory receptors (PD-1 and KIR2DL2/DL3) was assessed on HD-NK cells based on TIGIT expression following coculture with CD15+ monocytes from CRC. Bars indicate MFI ± SEM of the indicated makers. Grey contours represent negative controls for the indicated markers. (**D**) IFN-γ production was assessed on PMA/Iono-stimulated NK cells upon coculture with CD15+ monocytes. Bars represent frequency ± SEM of IFN-γ+ NK cells (n = 5), ** *p* < 0.01. (**E**) CD107a expression and IFN-γ production by NK cells following coculture with CD15+ monocytes in the presence or absence of NKp30 blocking mAb. Bars indicate percentage ± SEM of CD107a+ and IFN-γ + NK cells. PMA/Iono-stimulated NK cells from HD were used as control (n = 5) * *p* < 0.05; ** *p* < 0.01.

It has been described that IL-10 plays a role in the induction of TIGIT in NK cells [28]. Analysis of the supernatant of LPS-stimulated CD15+ monocytes isolated from CRC pts revealed that this population secretes a larger amount of IL-10 compared to CD15neg monocytes (Figure 4A).

Remarkably, following coculture between CD15+ monocytes and NK cells, the frequency of TIGIT+ NK cells significantly increased (Figure 4B), and the addition of a neutralizing antibody against IL-10 reduced this effect (Figure 4B). The subsequent analysis of NK cells revealed a downregulation of activating receptors, such as NKp30, NKp46 and NKG2D (Figure 4C), an upregulation of inhibitory receptors (PD-1 and KIR2DL2/DL3) and impaired functionality (Figure 4D).

It has been reported that the MDSC-mediated suppression of NK cells could also rely on cell contact through the NKp30 receptor [29]. We, thus, asked whether this new population of M-MDCSs might exert this suppressive mechanism by coculturing NK cells with IL-2 and CD15+ monocytes in the absence or presence of anti-NKp30 blocking antibody. IFN-γ and CD107a measurement revealed that blocking NKp30 substantially increased the frequency of IFN-γ+ and CD107a+ NK cells (Figure 4E), indicating that the inhibitory function of CD15+ monocytes was also dependent on NKp30 engagement.

Overall, these data suggest that CD15+ monocytes could suppress NK cell activity by promoting the acquisition of the inhibitory receptor TIGIT and through the engagement of NKp30.

### 2.4. TIGIT Expression on NK Cells Reduces Their Antitumor Ability

**Figure 5:**
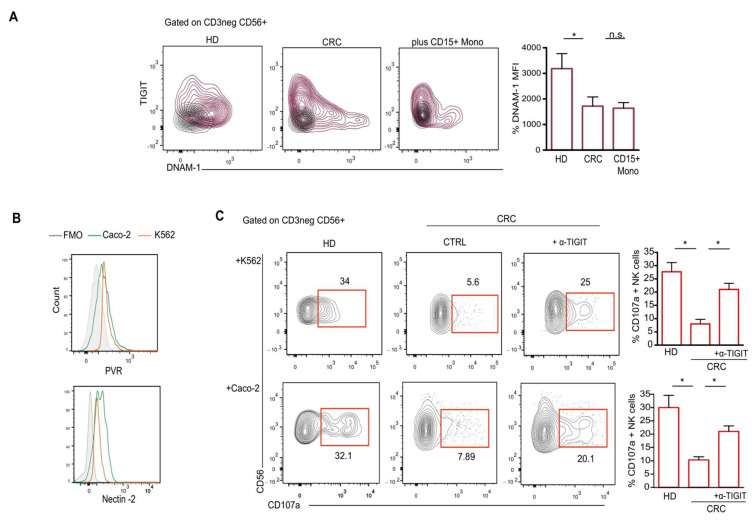
TIGIT limits NK cell-mediated tumor recognition in CRC pts. (**A**) Representative dot plots showing expression of TIGIT and DNAM-1 on NK cells from HD, CRC pts, or following coculture with CD15+ monocytes. Bars represent MFI ± SEM of DNAM-1 * *p* < 0.05. (**B**) Representative histograms showing the expression of PVR and Nectin-2 on Caco-2 and K562 cell line. FMO was used as control. (**C**) Representative dot plots and relative statistical analysis showing the expression of CD107a on NK cells from CRC pts following 6h of degranulation assay against K562 cells and Caco-2 cells in the presence or absence of TIGIT blocking mAb. Human IgG1 kappa isotype was used as control. Bars indicate percentage ± SEM of CD107a +NK cells (n = 3) * *p* < 0.05. n.s.= not significant.

TIGIT is the co-inhibitory counterpart of the DNAM-1 receptor [30]. Mechanistically, these receptors with opposite effects compete to bind the same ligands, PVR and Nectin-2, which are highly expressed on tumor cells [31,32]. Our data revealed that the acquisition of TIGIT observed on NK cells from CRC pts and upon coculture with CD15+ monocytes is accompanied by a concomitant downregulation of DNAM-1 expression (Figure 5A) and other activating receptors, such as NKp46, NKp30 and NKG2D (Figure S1 and Figure 4C), thus suggesting an impairment in tumor cell recognition and killing. Therefore, in order to assess the antitumoral functions of NK cells derived from CRC pts, we performed a degranulation assay against the classical target cells of NK cells, K562 and human colon adenocarcinoma cell line (Caco-2), both expressing PVR and Nectin-2 (Figure 5B). Our data revealed that, compared to NK cells from HD, NK cells from CRC pts failed in killing tumor cells (Figure 5C). TIGIT blockade was able to partially restore NK cell cytotoxicity (Figure 5C) [28]. These results indicate that the acquisition of TIGIT on the NK cells of CRC pts can directly reduce their capability to recognize and kill tumor cells.

## 3. Discussion

MDSC expansion is evident in the circulation and tumor microenvironment of many solid tumors, including CRC [33]. Some studies have reported that human CRC harbors a large population of G-MDSCs [34]. However, there are also other reports showing an increased level of both granulocytic and monocytic MDSC populations [35,36].

Here, by applying a gating strategy for M-MDSCs, we observed an increase in a newly described subpopulation characterized by the expression of the granulocyte marker CD15 in both the PB and tumor tissue of CRC pts. Along with CD15, these monocytic cells express other pan-neutrophil markers, such as CD66b, CD62L and MPO, and their frequency is correlated with the frequency of neutrophils. Remarkably, hematopoietic precursors exhibited myeloid bias with a significant increase in the level of circulating GMPs, suggesting, as a whole, a skewing toward granulocytic differentiation [17].

What remains to be understood is whether this neutrophil-like monocyte population might represent an immature population along G-MDSC differentiation and whether it is developmentally related to MPLGs. Similar to the neutrophil-to-lymphocyte ratio, in CRC pts, we found an increase in CD15+ monocytes over lymphocytes. It would be interesting to assess whether, in CRC pts, the frequency of CD15+ monocytes and their ratio with lymphocytes might correlate with patients’ clinical outcomes and thus represent a novel prognostic/predictive marker. Indeed, the potential positioning of these cells along G-MDSC differentiation could make them a more sensitive marker of an increase in G-MDSCs in CRC pts.

MDSCs are also considered a prognostic marker that can be used to predict therapy response to ICI [37,38]. Interestingly, in non-responder pts with advanced melanoma, among the MDSC subpopulation, CD15+ monocytes showed the highest increase [15], suggesting that this population could play a crucial role in ICI therapy resistance. Supporting this hypothesis, the reduction in CD15+ monocytes, observed in pts undergoing IFN-α or cytokine therapy prior to ICI, is associated with clinical responses [15]. The negative correlation between CD15+ monocytes and lymphocytes observed in these pts should support their immunosuppressive role [15].

The mechanisms through which MDSCs negatively regulate immune cells are extensively characterized [5,6,7,8]. Regarding the suppressive features of CD15+ monocytes, their capability to produce ROS and iNOS has been reported [14]. However, their immunoregulatory activity in terms of interaction with components of the immune system remains unknown. In this study, we evaluated the interaction between CD15+ monocytes and NK cells and revealed that this new group of MDSCs can promote, in NK cells, the acquisition of TIGIT expression with concomitant downregulation of its activating counterpart DNAM-1 through the engagement of NKp30. High TIGIT expression was also observed in CRC pts and resulted in the impairment of NK cell effector functions, which was partially restored upon anti-TIGIT treatment. Accordingly, it has been reported that TIGIT, but not other immune checkpoint molecules such as CTLA-4 and PD-1, is associated with NK cell exhaustion in tumor-bearing mice and pts with CRC [28] and that the blockade of TIGIT restored NK cell antitumor activity [39,40].

Based on our observations, another possible approach to prevent NK cell dysfunction is to target CD15+ monocytes. Targeting MDSCs could be exploited in different ways, including the inhibition of MDSC recruitment, differentiation, and function [41]. The high frequency of CD15+ monocytes observed in both circulation and tumor tissue might suggest the active recruitment of these cells from the PB to tumor tissues. This notion was supported by the evidence that tumor-infiltrating myeloid precursors can upregulate CXCR4, a major homing receptor for the chemokine SDF-1, to recruit and retain them in tumor tissues [42]. In the case of CD15+ monocytes, further investigations are needed to establish, and potentially target, the chemokine receptors able to guide the homing of these cells within tumor tissues. Alternatively, the blockade of tumor-derived factors such as IL-6, G-MCS and GM-CSF, involved in the differentiation process from GMPs/MLPGs [17,18] but also in the acquisition of suppressive features, could represent a plausible approach to limit CD15+ monocyte accumulation.

Finally, we found that CD15+ monocytes constitute a subpopulation of MDSCs in CRC pts that exhibit granulocyte features and interfere with NK cell antitumor responses. Further investigations are needed to increase our knowledge about the pathways of the development, immunosuppressive activity and clinical relevance of these cells in various tumors.

## 4. Materials and Methods

### 4.1. Sample Collection

Colorectal carcinoma tissues (tumor and healthy mucosa, the latter collected at a distance of 10 cm from primary tumor) and whole blood samples of 30 CRC pts (range 52–83 years) were collected from the General Surgery and Surgical Oncology Unit of the University Hospital “G. Martino”, Messina, Italy. Patients’ baseline characteristics are summarized in Supplementary Table S1. Whole blood of HD (range 30–60 years) was obtained from the Unit of Transfusion Medicine and used as the control. All samples were collected after obtaining informed consent. This study was performed in accordance with the Declaration of Helsinki and was approved by the Ethical Committee of the University Hospital Policlinico G. Martino, Messina, Italy (Protocol ID. 55-23 on 20 March 2023)

### 4.2. Cell Isolation and Phenotypical Analysis

Tissues from CRC pts were processed as previously described [43]. Briefly, tissues were extensively washed in phosphate-buffered saline solution (PBS) to remove red blood cells, debris and clots, mechanically minced by scissors to obtain small fragments and then digested in RPMI, supplied with a cocktail of EDTA (1 mM), DL-Dithiothreitol DTT (1 mM) and FBS (1%), at room temperature in an agitator for 20 min in order to detach lamina propria lymphocytes. Samples were then enzymatically digested with a cocktail containing collagenase IV (1 mg ml^−1^) and DNAse (100 μg ml^−1^) in complete RPMI (Pen/Strep and Glutamine) for 45 min at 37 °C 5% CO_2_. Subsequently, cell suspensions were filtered and washed by centrifugation to remove residual enzymes.

To obtain mononuclear cells from tissue suspensions and from whole blood, samples were centrifuged by Ficoll-Hypaque (Sigma-Aldrich, Burlington, MA, USA) density gradient at 2100 RPM for 45 min at room temperature.

For identification and isolation of cell subsets, the following gating strategies were used: M-MDSCs as CD45+LINneg (CD3, CD19, CD56) HLA-DRlow/neg CD11b+CD33+ CD14+CD15+/neg; monocytes as CD14+CD15negHLA-DR+; neutrophils as CD15+CD14neg; and NK cells as CD3neg CD56+.

Alternatively, 200 ul of whole blood from CRC pts was stained with specific mAbs for 10 min in the dark at room temperature, and then erythrocytes were lysed using lysing solution (BD FACS™ Lysing Solution) for 20 min and finally washed twice in PBS.

For hematoxylin and eosin staining, CD15+, CD15neg monocytes and neutrophils were first sorted via FACS from whole blood following lysis of erythrocytes, as abovementioned, and then air-dried in chamber slides (Thermofisher, Waltham, MA, USA), fixed in 4% paraformaldehyde, washed in PBS and stained in hematoxylin for 2 min. Then, cells were washed in running tap water and counterstained for 2 min with eosin, washed again in distilled water and observed at a final magnification 100× (Eclipse Ci, Nikon Europe B.V., Amstelveen, The Netherlands).

For surface analysis, cells were stained with specific mAbs for 15 min at room temperature and then PBS-washed. Alternatively, cells were surface-stained for 30 min at room temperature with the following supernatants: anti-PVR (L95 IGg1) or anti-Nectin-2 (L14 IGg1). FMO was used as the staining control.

For IFN-γ or MPO intracellular staining, cells were surface-stained and then fixed in 1% paraformaldehyde for 20 min on ice in the dark, permeabilized with saponin 0.1% in PBS and stained with anti- IFN-γ or anti-MPO mAbs for 45 min at room temperature and washed twice with PBS before the flow cytometric analysis.

### 4.3. Cell Culture Assay

Human CRC cell line Caco-2 (ATCC HTB-37 ™) and K562 (ATCC CCL-243 ™) were cultured in Dulbecco’s modified Eagle’s complete medium (DMEM) and RPMI, respectively, at 37 °C in a humidified incubator with 5% CO2. Cells were grown until confluence and detached using 0.25% trypsin and 0.02 mol/l EDTA in PBS for analysis of PVR and Nectin-2 expression.

IFN-γ production or CD107a expression were evaluated on NK cells freshly isolated from PB of CRC pts or on NK cells from HD upon coculture with CD15+ monocytes, stimulated for 4h with PMA/Ionomycin (10 ug/mL and 1 ug/mL, respectively; Sigma-Aldrich). Alternatively, IL-2-stimulated NK cells from HD were cocultured with CD15+ monocytes from CRC pts in the presence of IL-10 blocking Ab (130-096-041, Miltenyi, Bergisch Gladbach, Cologne, Germany) or NKp30 blocking mAb (F252 IgM). In each experimental condition, monensin and brefeldin (2 μmol/L and 10 μg/mL, respectively; Sigma-Aldrich) were added for the last 3 h, as previously described [44].

Alternatively, CD107a expression was evaluated on NK cells, sorted by FACS from PB of CRC pts and HD following coculture with Caco-2 or K562 target cells at a 2:1 ratio (E:T). Where indicated, 5 μg/mL of anti-TIGIT antibody (Tiragolumab Cat. No.: HY-P9986) or corresponding human IgG1 kappa isotype control (Cat. No.: HY-P99001) were added throughout the experiments.

The expression of activating and inhibitory receptors was evaluated on NK cells isolated from the PB of CRC pts and HD, or on NK cells from HD following 3 days of coculture with CD15+ monocytes.

### 4.4. Imaging Flow Cytometry

CD15+ monocytes, CD15neg monocytes or neutrophils from whole blood of CRC pts were fixed with 2% paraformaldehyde (PFA), stained with mAbs and nuclear dye DAPI and then analyzed by an Imagestream cytometer as previously described [45]. Cells were analyzed using the Amnis ImageStream X Mark II flow cytometer. After acquisition of focused cells and application of a compensation matrix, based on single-stained controls, cells were analyzed with IDEAS 3.0 software. Images were shown as single cells, comparing the profile of Brightfield (BF, Ch01), CD14 (Ch11), CD15 (Ch02), DAPI (Ch07) and channel merge.

### 4.5. Enzyme-Linked Immunosorbent Assay

The concentration of IL-10 was measured on culture supernatants of CD15+ and CD15neg monocytes isolated from the PB of CRC pts stimulated with LPS (1 ug/mL, Sigma) for 24 h using validated commercial ELISA kits, according to the manufacturer’s instructions (Human IL-10 Standard ABTS ELISA Development Kit Catalog# 900-K21, Peprotech). Unstimulated monocytes were used as the control.

### 4.6. Flow Cytometry

The mAbs used in this study are summarized in Table 1. Sample acquisition was performed on FACSCantoII or FACSymphony (BD Biosciences, Franklin Lakes, NJ, USA) flow cytometers and cell sorting was performed on FACSAria II cell sorter (BD Biosciences). Data were acquired by FACS Diva (BD Biosciences) and analyzed by FlowJo version VX (Tree Star Inc, Oakland, CA, USA) software.

### 4.7. Statistical Analysis

Depending on the data, either a paired Student’s *t*-test or an ANOVA test was applied to evaluate statistical significance. *p*-values lower than 0.05 were considered statistically significant (* *p* < 0.05; ** *p* < 0.01; *** *p* < 0.001). Statistics were calculated using GraphPad Prism 4 software.

## Figures and Tables

**Table 1:** List of antibodies used in the study.

mAbs	Clone	Fluorochrome	Distributors
CD3	UCHT1	FITC	Beckman Coulter (Brea, CA, USA)
CD19	J3.119	FITC	Beckman Coulter
CD45	J.33	Krome Orange	Beckman Coulter
CD14	RMO52	APC-Alexa Fluor 700	Beckman Coulter
CD11b	Bear1	APC	Beckman Coulter
CD33	D3HL60.251	PC5	Beckman Coulter
HLA-DR	Immu-357	PC7	Beckman Coulter
CD15	80H5	FITC	Beckman Coulter
CD62L	DREG56	PE	Beckman Coulter
CD66b	80H3	APC-Alex Fluor 750	Beckman Coulter
MPO	5B8	PE	BD Biosciences
CD49d	HP2/1	APC	Beckman Coulter
CD16	3G8	Pacific Blue	Beckman Coulter
CD45RA	2H4LDH11LDB9 (2H4)	ECD	Beckman Coulter
CD123	SSDCLY107D2	PC5.5	Beckman Coulter
CD56	N901 (NKH-1)	PC7	Beckman Coulter
IFN-γ	45	FITC	Beckman Coulter
CD107a	H4-A3	Pacific Blue	Beckman Coulter
TIGIT	741182	BUV395	BD Biosciences
DNAM-1	11A8	BV605	BD Biosciences
CD34	581	ECD	Beckman Coulter
CD38	LS198-4-3	APC-AlexaFluor 700	Beckman Coulter
Dapi	n.a.	n.a.	ThermoFisher
NKp30	Z25	PE	Beckman Coulter
NKp46	BAB281	PC5	Beckman Coulter
NKG2D	ON72	APC	Beckman Coulter
KIR(KIR2DL2/DL3)	DX27	FITC	Miltenyi Biotec
PD-1	PD1.3	PC7	Beckman Coulter
Anti-IgG1	n.a.	APC-AlexaFluor 488	Invitrogen (Waltham, MA USA)

n.a. = not available.

## Data Availability

Data are contained within the article or Supplementary Materials.

## Supplementary Materials

The following supporting information can be downloaded at: https://www.mdpi.com/article/10.3390/ijms25158470/s1.
