# Supplementary material for: Neutrophil-like Monocytes Increase in Patients with Colon Cancer and Induce Dysfunctional TIGIT+ NK Cells
Source: Int J Mol Sci. 2024 Aug 2;25(15):8470. doi: 10.3390/ijms25158470 (PMC11313383; doi:10.3390/ijms25158470)
Supplement: Supplementary file 1 [file ijms-25-08470-s001.zip › ijms-3125199-supplementary.pdf]

Supplemental Materials

Neutrophil-like monocytes increase in patients with colon cancer and induce dysfunctional TIGIT+ NK cells

Alessia Calabrò [1#], Fabiana Drommi [1#], Giacomo Sidoti Migliore [2], Gaetana Pezzino [1], Grazia Vento [3], Josè Freni [4], Gregorio Costa [1,5], Riccardo Cavaliere [5], Irene Bonaccorsi [1,5], Mariagrazia Sionne [6], Stefania Nigro [6], Giuseppe Navarra [6], Guido Ferlazzo [3,7,*], Claudia De Pasquale [1,†] and Stefania Campana [1,†]

[1] Laboratory of Immunology and Biotherapy, Department Human Pathology "G. Barresi", University of Messina, Via Consolare Valeria 1, 98125, Messina, Italy

[2] Translational Immunobiology Unit, Laboratory of Infectious Diseases, National Institute of Allergy and Infectious Diseases, National Institutes of Health, BLDG 50, RM 6308, Bethesda, MD 20892, USA

[3] Department of Experimental Medicine (DIMES), University of Genoa, Via Leon Battista Alberti 2, 16132 Genova, Italy

[4] Laboratory of Histology, Department of Biomedical, Dental, Morphological and Functional Imaging Sciences, University of Messina, Via Consolare Valeria 1, 98125 Messina, Italy

[5] Clinical Pathology Unit, University Hospital Policlinico G. Martino, 98125, Messina, Italy

[6] Oncologic Surgery, Department of Human Pathology of Adult and Evolutive Age, University of Messina, Via Consolare Valeria 1, 98125 Messina, Italy

[7] Unit of Experimental Pathology and Immunology, IRCCS Ospedale Policlinico San Martino, Largo R. Benzi 10, 16132 Genova, Italy.

# These authors contributed equally to this work.
† These authors contributed equally to this work.

* Correspondence: guido.ferlazzo@unige.it (G.F.)

.

Table S1. Patient's baseline characteristics and CD15 expression on monocytes.

Patient n°	Age, y/sex	Clinical stage (ISS)	CD15 expression on monocytes
1	82/M	I	+
2	56/M	IV	+++
3	78/M	II	++
4	67/M	I	+
5	52/M	I	+
6	70/M	III	++
7	64/F	II	++
8	81/M	III	+++
9	79/F	II	+
10	75/F	I	+
11	78/M	IV	++
12	83/F	III	++
13	79/F	II	++
14	81/M	IV	+++
15	77/F	IV	+++
16	80/F	III	++
17	62/M	II	+
18	72/F	I	+
19	74/M	I	+
20	57/F	IV	+++
21	78/M	IV	+++
22	77/M	III	++
23	82/M	II	+
24	73/M	IV	+++
25	75/M	III	++
26	83/F	IV	+++
27	71/F	II	++
28	64/F	III	++
29	78/M	IV	+++
30	73/F	IV	+++

+, CD15+ Monocytes (20%-30%); ++, CD15+ Monocytes (30%-40%); +++, CD15+ Monocytes (>40%)F, female; M, male; ISS, International Staging System.

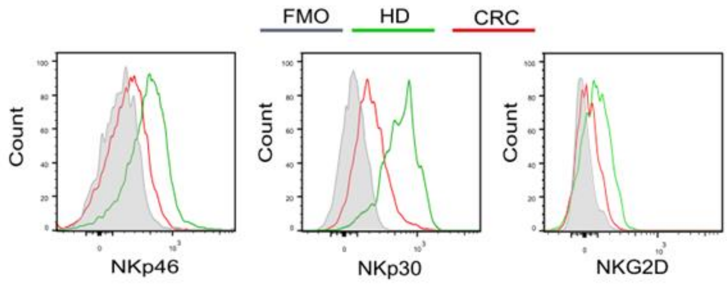

Figure S1. NK cells from CRC pts display reduced expression of activating receptors.
Expression of NKp30, NKp46 and NKG2D was assessed on NK cells from CRC pts and HD by flow cytometry. FMO was used as control.
